# Concomitant Traumatic Brain Injury Exacerbates Endotheliopathy in Patients with Spinal Cord Injury

**DOI:** 10.1177/2689288X251377022

**Published:** 2025-09-22

**Authors:** Shahab Hafezi, Miguel A. Ruiz-Cardozo, Sarbani Ghosh, Sravanthi Bandla, Matthew N. Montoya Rush, Anand Dharmarajan, Mark H. Hoofnagle, Isaiah R. Turnbull, Camilo A. Molina, Grace M. Niziolek

**Affiliations:** ^1^ Section of Acute and Critical Care Surgery, Department of Surgery, Washington University School of Medicine in St. Louis, Saint Louis, Missouri, USA.; ^2^ Department of Neurosurgery, Washington University School of Medicine in St. Louis, Saint Louis, Missouri, USA.

**Keywords:** biomarker, endotheliopathy, inflammation, neurotrauma, spinal cord injury, traumatic brain injury

## Abstract

Neurotrauma can cause endothelial dysfunction, characterized by neurovascular barrier disruption, tissue edema, neuroinflammation, and coagulation abnormalities, all of which may contribute to secondary injuries and worsened clinical outcomes. Here, we assess the effect of different types of neurotrauma on the local levels of biomarkers of endothelial injury and inflammation. Cerebrospinal fluid (CSF) samples were collected at multiple time points from patients with isolated traumatic spinal cord injury (SCI) and patients with concomitant SCI and traumatic brain injury (TBI). CSF levels of analytes associated with endothelial damage, as well as inflammatory mediators, were measured. Compared with patients with isolated SCI, those with SCI + TBI demonstrated significantly elevated CSF levels of multiple biomarkers linked to endotheliopathy and inflammation. In the presence of TBI, the highest increases in CSF levels of endothelial markers were observed for matrix metalloproteinase 10 (MMP-10), vascular endothelial growth factor A (VEGF-A), and fibroblast growth factor 2 (FGF-2). Among inflammatory factors, thymic stromal lymphopoietin (TSLP) showed the most pronounced difference in CSF content in patients with SCI + TBI compared with those with SCI alone, followed by interferon α2 (IFNα2) and granulocyte-macrophage colony-stimulating factor (GM-CSF). Interestingly, CSF levels of MMP-1, MMP-10, VEGF-A, IFNα2, and TSLP significantly correlated with injury severity score. Our findings indicate that, in the presence of concomitant TBI, patients with SCI exhibit higher CSF levels of biomarkers associated with endotheliopathy, blood–brain barrier breakdown, protease-mediated degradation of endothelial glycocalyx, and neuroinflammation. These results identify potential theranostic biomarkers to stratify high-risk patients and mitigate neurovascular damage, thereby improving clinical outcomes.

## Introduction

Neurotrauma, including traumatic brain injury (TBI) and spinal cord injury (SCI), affects approximately 30 million individuals globally each year, imposes substantial costs and challenges to health care, and is a leading cause of mortality and long-term morbidity following injury.^1^ TBI is the second most common cause of death after major trauma and puts patients at increased risk for in-hospital complications.^2–5^ Long-term morbidities associated with neurotrauma include motor and cognitive deficits, seizures, neurodegeneration, dementia, and psychiatric disorders.^6^

Endothelial integrity is vital for vascular homeostasis and the regulation of biological processes, including vascular permeability, coagulation, and inflammation.^7^ Disruption of the endothelial integrity of the central nervous system (CNS) following neurotrauma can lead to neurovascular barrier disruption, neural tissue edema, coagulopathy, and cytokine-mediated inflammatory response, ultimately resulting in secondary brain or spinal cord injuries.^8,9^ Therefore, preventing or mitigating the consequences of endothelial dysfunction in the CNS represents a potential therapeutic target to improve clinical outcomes in patients with neurotrauma.

Despite extensive research in this area, the impact of the type of neurotrauma on endothelial dysfunction remains poorly understood. In addition, the most commonly used cerebrospinal fluid (CSF) biomarkers of neurotrauma, such as glial fibrillary acidic protein, tau protein, and S-100β, are not indicative of the presence of endothelial dysfunction.^10,11^ Therefore, we aimed to assess the effect of different types of neurotrauma on the local milieu of biomarkers related to endothelial dysfunction. We hypothesized that biomarkers associated with endothelial injury and dysfunction, glycocalyx degradation, neurovascular barrier breakdown, and neuroinflammation would be found at higher levels when TBI is present.

## Materials and Methods

**FIG. 1.**
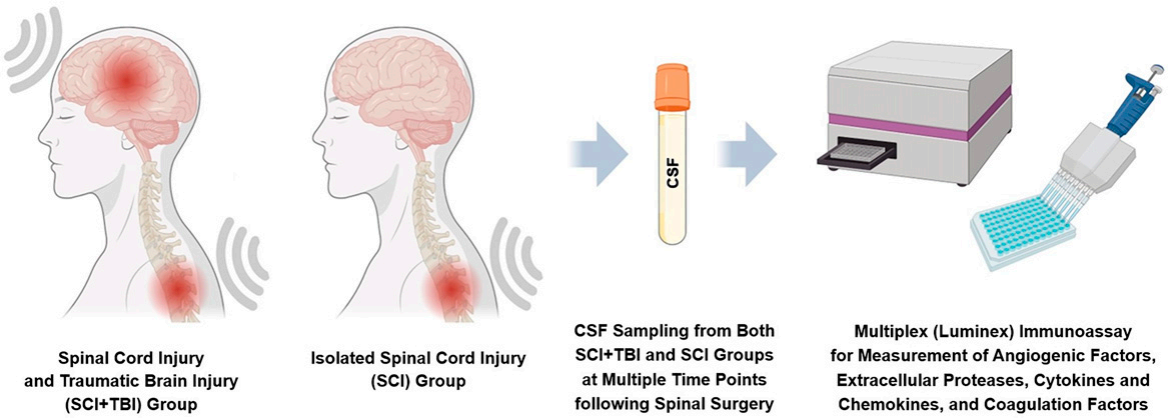
CSF was collected from patients with SCI + TBI and patients with isolated SCI at multiple time points post-spinal surgery, and CSF levels of markers for endothelial injury, blood-brain barrier disruption, endothelial glycocalyx degradation, neuroinflammation, and coagulation disturbance were measured and analyzed. CSF, cerebrospinal fluid; SCI, spinal cord injury; TBI, traumatic brain injury.

This is a retrospective observational study using CSF collected from patients with neurotrauma at an urban Level 1 Trauma Center. The study was approved by the Washington University in St. Louis Institutional Review Board and was conducted in accordance with the Declaration of Helsinki. Two patients with isolated SCI and three patients with SCI + TBI were included. In all patients, spinal surgery was performed within 24 h of the traumatic event. CSF samples were obtained postoperatively at multiple time points (24, 36, 48, 60, 72, 84, and 96 h) via lumbar drains (Fig. 1). Lumbar drains were placed intraoperatively as part of standard care and based on clinical indications, and serial CSF samples were collected only from patients in whom the drains were functional and there was no evidence of impaired CSF flow.

A Milliplex Plexpedition 115-plex premixed kit (HPLX1-115SP-PX, MilliporeSigma) was used to measure a total of 115 analytes. Markers associated with endothelial injury or activation, protease-mediated breakdown of the endothelial glycocalyx, blood–brain barrier (BBB) or blood–spinal cord barrier (BSCB) disruption (endothelial markers), as well as cytokines and chemokines (inflammatory markers), were analyzed. Markers were included in the analysis if the concentration readings fell within the standard curve and fell within the detectable range of the assay, and if all five patients had complete results for at least four time points out of seven. Endothelial markers were erythropoietin (EPO), fibroblast growth factor 2 (FGF-2), heparin-binding epidermal growth factor-like growth factor (HB-EGF), hepatocyte growth factor (HGF), interleukin 8 (IL-8), matrix metalloproteinase 1 (MMP-1), MMP-2, MMP-3, MMP-9, MMP-10, platelet-derived growth factor AA (PDGF-AA), vascular endothelial growth factor A (VEGF-A), VEGF-C, and VEGF-D. Inflammatory markers included fms-like tyrosine kinase 3 ligand (FLT3 ligand), granulocyte-macrophage colony-stimulating factor (GM-CSF), I-309, interferon α2 (IFNα2), IFNγ, interleukin 1 receptor antagonist (IL-1Ra), IL-4, IL-5, IL-6, IL-8, IL-9, IL-10, IL-15, IL-18, interferon gamma inducible protein 10 (IP-10), macrophage colony-stimulating factor (M-CSF), macrophage inflammatory protein 3β (MIP-3β), monocyte chemoattractant protein 1 (MCP-1), myostatin, osteoprotegerin (OPG), soluble Fas (sFas), transforming growth factor α (TGFα), tumor necrosis factor-related apoptosis-inducing ligand (TRAIL), and thymic stromal lymphopoietin (TSLP).

The CSF samples collected at 24 and 36 h post-spinal surgery were also tested for common coagulation markers with a separate human Luminex multiplex assay (LXSAHM-11, R&D Systems). Coagulation markers consisted of a disintegrin and metalloproteinase with thrombospondin motifs 13 (ADAMTS13), D-dimer, P-selectin, thrombomodulin, tissue factor, and urokinase-type plasminogen activator (uPA), also known as urokinase.

### Statistical analysis

GraphPad Prism (Windows) was used to analyze the data and plot the graphs. Figure illustrations were created with BioRender.com. To generate a heatmap, Z-scores for each time point in each group were calculated using the following formula:

Z−score = (Mean of Biomarker among Patients of Either Group – Mean of Biomarker among All Patients)/Standard Deviation of Biomarker among All Patients

Principal component analysis (PCA) was performed using the Phantasus platform.^12^ Data are expressed as median and interquartile range, and values were compared between the two groups using the Mann–Whitney *U* test. Biomarker readings from all time points were pooled for each patient group. Correlations between injury severity score (ISS) and biomarker levels were evaluated. A *p*-value < 0.05 was considered statistically significant. In addition, the average relative change (in percentage) in the CSF marker levels in the SCI + TBI group compared with the SCI group was determined for markers that exhibited significant differences between the two groups. First, to obtain a mean for each patient, values from all time points were averaged. Then, we derived a mean for each group by averaging the mean values of the patients in that group. Finally, the following formula was used to calculate the average relative change:

Average Relative Change (%) = [(Mean ‘SCI and TBI’ – Mean ‘SCI’)/Mean ‘SCI’] × 100

## Results

**FIG. 2.**
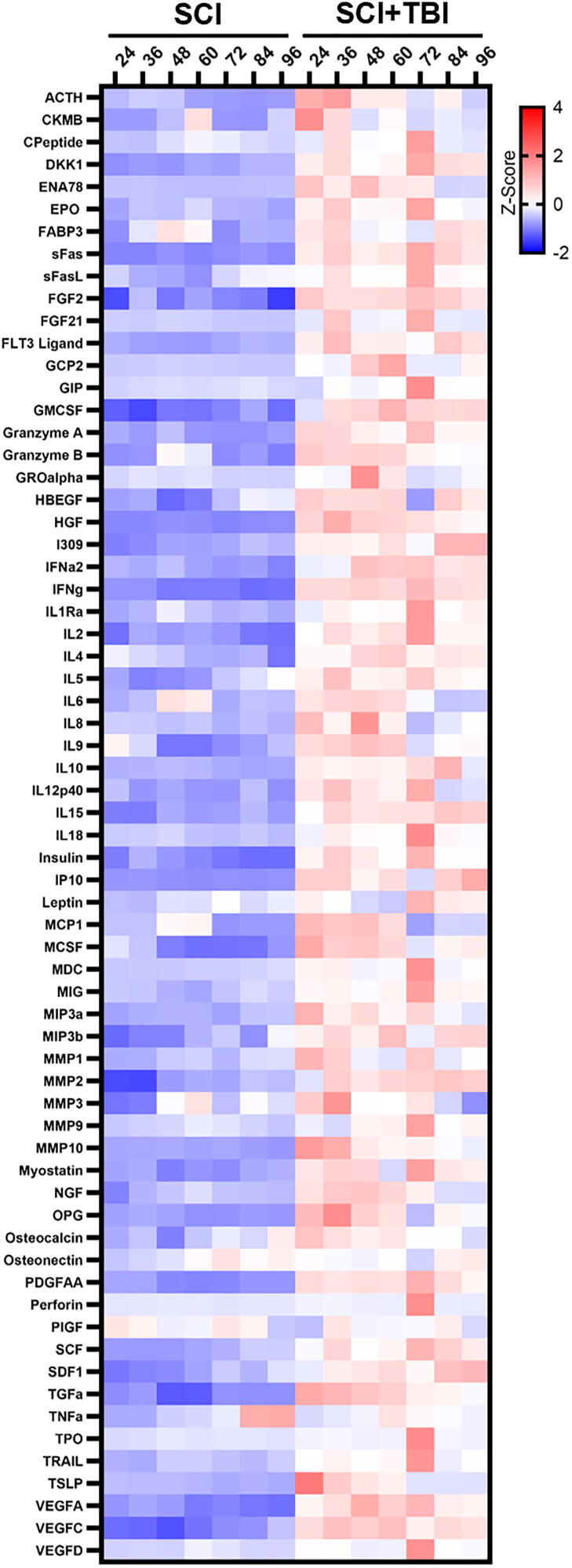
Heatmap of CSF biomarkers Z-scores in SCI and patients with SCI + TBI over time. CSF, cerebrospinal fluid; SCI, spinal cord injury; TBI, traumatic brain injury.

**FIG. 3.**
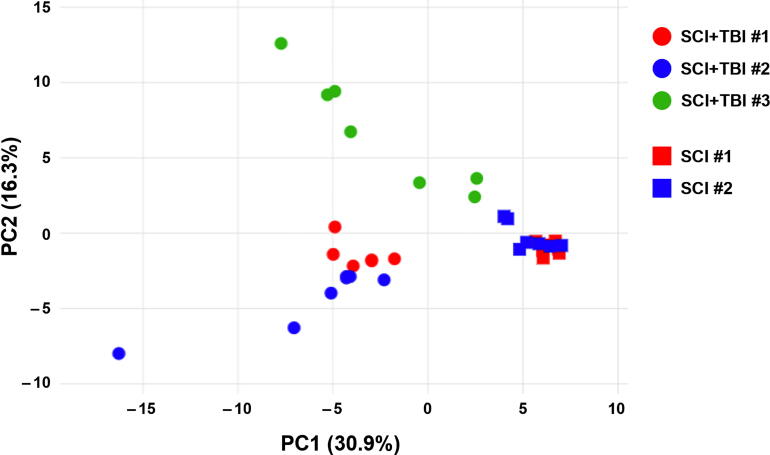
Principal component analysis of the individual patients included in the study. PC, principal component; SCI, spinal cord injury; TBI, traumatic brain injury.

Patient demographics, clinical characteristics, and injury severity are summarized in Table 1. Heatmap analysis of all 115 analytes demonstrated differences in CSF levels of various markers, including those analyzed in this study, between patients with isolated SCI and those with SCI + TBI, with no notable changes over time (Fig. 2). In addition, to evaluate the dispersion of the data and identify the main sources of variability, dimensionality reduction was performed by PCA, which revealed that the first two components accounted for 47% of the variance (Fig. 3). PCA showed that samples from cases with isolated SCI formed a tight cluster, indicating minimal heterogeneity across individuals and over time. In contrast, samples from patients with SCI + TBI exhibited much greater variance, contributing to the majority of the observed dispersion across all samples. In fact, SCI + TBI subjects were distinct and segregated from both SCI and other SCI + TBI subjects.

**Table 1.** Patient Demographics and Clinical Characteristics

Injury pattern	Age	Sex	Mechanism of injury	ISS	Head AIS	Neck AIS	Type of TBI
TBI + SCI	58	F	Dragged by a horse	21	4	4	Subarachnoid hemorrhage Subdural hematoma Intraparenchymal hemorrhage
TBI + SCI	47	F	Motor vehicle crash	17	2	4	Concussion
TBI + SCI	37	F	Motor vehicle crash	38	3	4	Subdural hematoma
SCI	25	M	Ground level fall	9	0	4	—
SCI	60	M	Motor vehicle crash	16	0	4	—

AIS, abbreviated injury scale; F, female; ISS, injury severity score; M, male; SCI, spinal cord injury; TBI, traumatic brain injury.

**FIG. 4.**
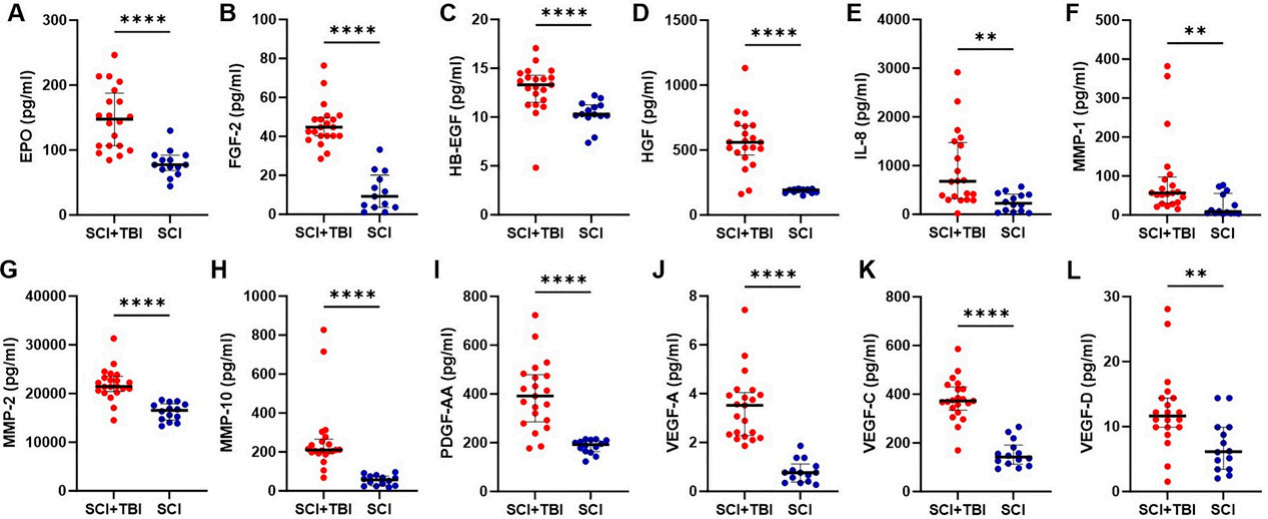
CSF levels of markers of endothelial injury, including EPO **(A)**, FGF-2 **(B)**, HB-EGF **(C)**, HGF **(D)**, IL-8 **(E)**, MMP-1 **(F)**, MMP-2 **(G)**, MMP-10 **(H)**, PDGF-AA **(I)**, VEGF-A **(J)**, VEGF-C **(K)**, and VEGF-D **(L)**. CSF, cerebrospinal fluid; EPO, erythropoietin; FGF, fibroblast growth factor; HB-EGF, heparin-binding epidermal growth factor-like growth factor; HGF, hepatocyte growth factor; IL-8, interleukin 8; MMP, matrix metalloproteinase; PDGF-AA, platelet-derived growth factor AA; SCI, spinal cord injury; TBI, traumatic brain injury; VEGF, vascular endothelial growth factor.

**FIG. 5.**
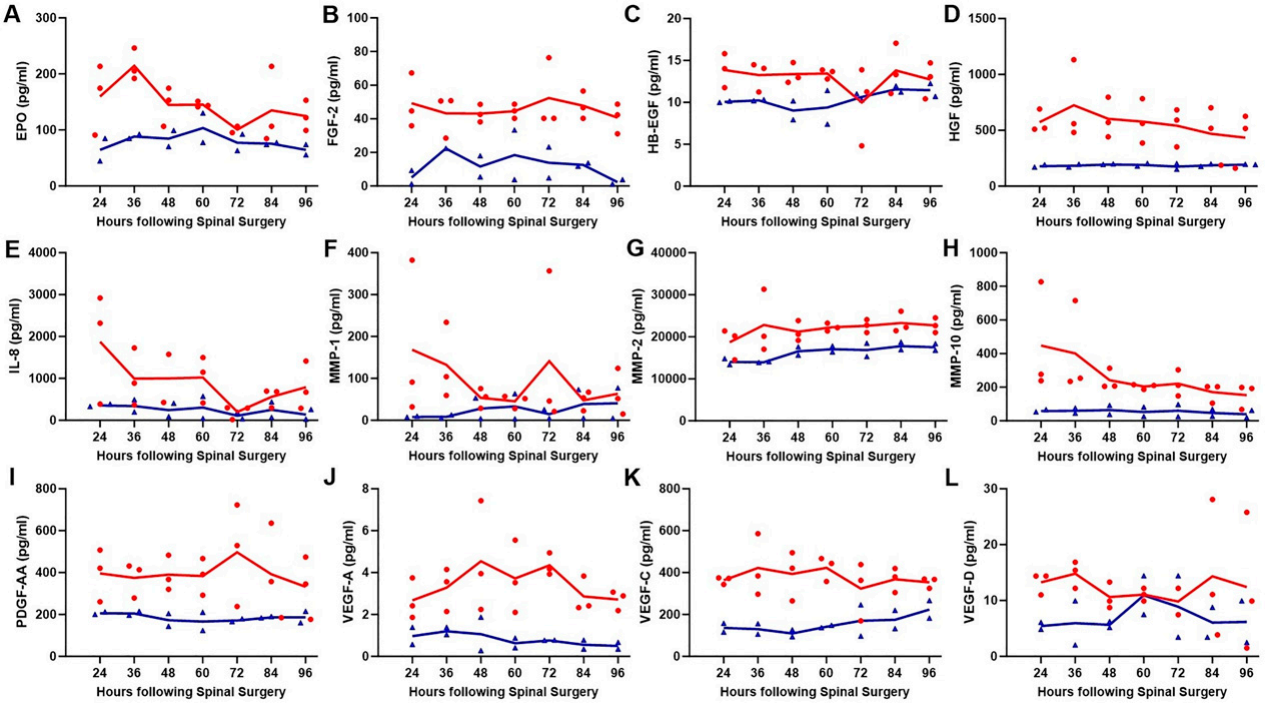
Changes over time in CSF levels of markers of endothelial injury, including EPO **(A)**, FGF-2 **(B)**, HB-EGF **(C)**, HGF **(D)**, IL-8 **(E)**, MMP-1 **(F)**, MMP-2 **(G)**, MMP-10 **(H)**, PDGF-AA **(I)**, VEGF-A **(J)**, VEGF-C **(K)**, and VEGF-D **(L)**. Each *red circle* (SCI + TBI) or *blue triangle* (SCI) represents a data point, with *red lines* (SCI + TBI) and *blue lines* (SCI) indicating the means. CSF, cerebrospinal fluid; EPO, erythropoietin; FGF, fibroblast growth factor; HB-EGF, heparin-binding epidermal growth factor-like growth factor; HGF, hepatocyte growth factor; IL-8, interleukin 8; MMP, matrix metalloproteinase; PDGF-AA, platelet-derived growth factor AA; SCI, spinal cord injury; TBI, traumatic brain injury; VEGF, vascular endothelial growth factor.

Compared with patients with isolated SCI, those with SCI + TBI had significantly elevated CSF levels of markers of endothelial injury (Fig. 4), including EPO (*p* < 0.0001; Fig. 4A), FGF-2 (*p* < 0.0001; Fig. 4B), HB-EGF (*p* < 0.0001; Fig. 4C), HGF (*p* < 0.0001; Fig. 4D), IL-8 (*p* < 0.01; Fig. 4E), MMP-1 (*p* < 0.01; Fig. 4F), MMP-2 (*p* < 0.0001; Fig. 4G), MMP-10 (*p* < 0.0001; Fig. 4H), PDGF-AA (*p* < 0.0001; Fig. 4I), VEGF-A (*p* < 0.0001; Fig. 4J), VEGF-C (*p* < 0.0001; Fig. 4K), and VEGF-D (*p* < 0.01; Fig. 4L). No significant differences were observed between the two cohorts when MMP-3 (*p* = 0.089) and MMP-9 (*p* = 0.066) were examined. Figure 5 demonstrates changes over time in CSF levels of significantly different endothelial markers, which remain consistently higher in patients with SCI + TBI.

**FIG. 6.**
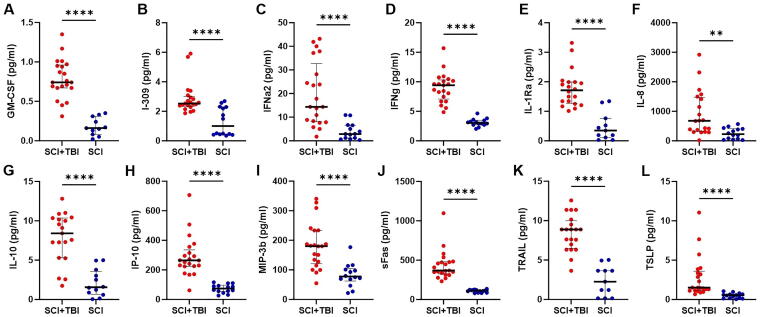
CSF levels of inflammatory markers, including GM-CSF **(A)**, I-309 **(B)**, IFNα2 **(C)**, IFNγ **(D)**, IL-1Ra **(E)**, IL-8 **(F)**, IL-10 **(G)**, IP-10 **(H)**, MIP-3β **(I)**, sFas **(J)**, TRAIL **(K)**, and TSLP **(L)**. CSF, cerebrospinal fluid; GM-CSF, granulocyte-macrophage colony-stimulating factor; IFN, interferon; IL-1Ra, interleukin-1 receptor antagonist; IL, interleukin; IP, interferon gamma inducible protein; MIP, macrophage inflammatory protein; SCI, spinal cord injury; sFas, soluble Fas; TBI, traumatic brain injury; TRAIL, tumor necrosis factor-related apoptosis-inducing ligand; TSLP, thymic stromal lymphopoietin.

**FIG. 7.**
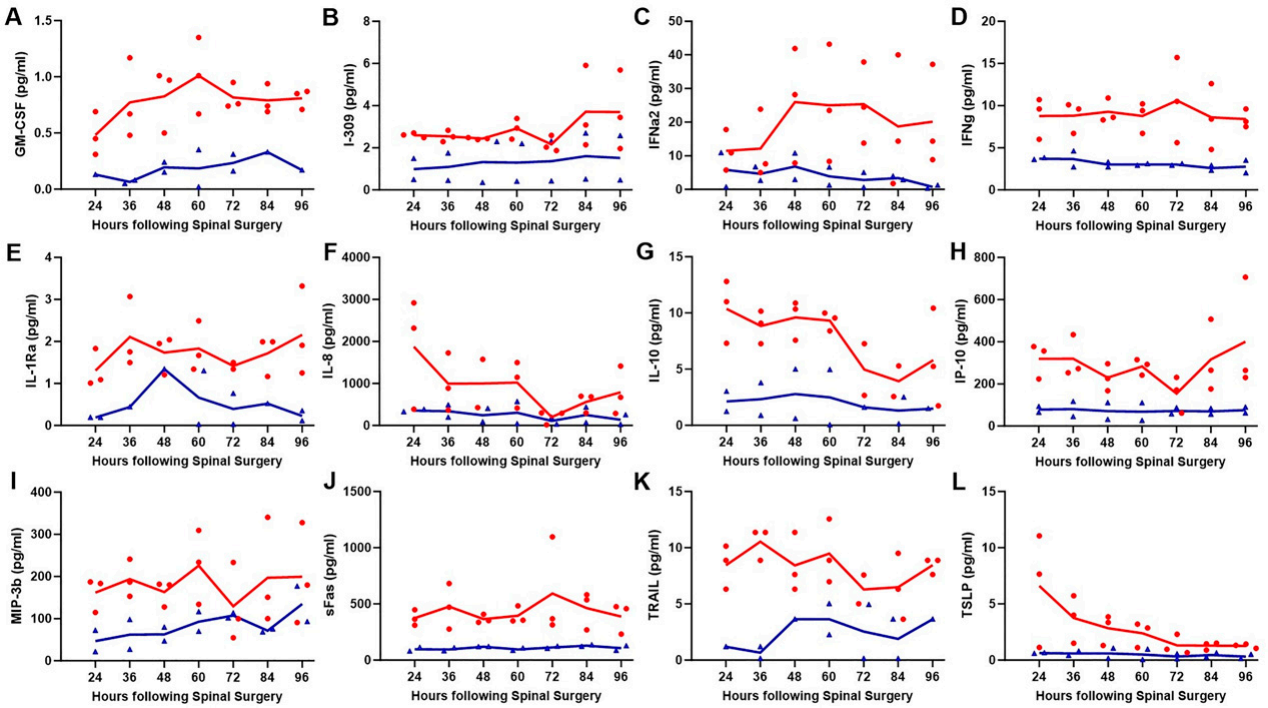
Changes over time in CSF levels of inflammatory markers, including GM-CSF **(A)**, I-309 **(B)**, IFNα2 **(C)**, IFNγ **(D)**, IL-1Ra **(E)**, IL-8 **(F)**, IL-10 **(G)**, IP-10 **(H)**, MIP-3β **(I)**, sFas **(J)**, TRAIL **(K)**, and TSLP **(L)**. Each *red circle* (SCI + TBI) or *blue triangle* (SCI) represents a data point, with *red lines* (SCI + TBI) and *blue lines* (SCI) indicating the means. CSF, cerebrospinal fluid; GM-CSF, granulocyte-macrophage colony-stimulating factor; IFN, interferon; IL-1Ra, interleukin-1 receptor antagonist; IL, interleukin; IP, interferon gamma inducible protein; MIP, macrophage inflammatory protein; SCI, spinal cord injury; sFas, soluble Fas; TBI, traumatic brain injury; TRAIL, tumor necrosis factor-related apoptosis-inducing ligand; TSLP, thymic stromal lymphopoietin.

Inflammatory cytokines and chemokines exhibited significantly elevated CSF levels in the SCI + TBI cohort compared with the SCI group (Fig. 6), including FLT3 ligand (*p* < 0.0001), GM-CSF (*p* < 0.0001; Fig. 6A), I-309 (*p* < 0.0001; Fig. 6B), IFNα2 (*p* < 0.0001; Fig. 6C), IFNγ (*p* < 0.0001; Fig. 6D), IL-1Ra (*p* < 0.0001; Fig. 6E), IL-10 (*p* < 0.0001; Fig. 6G), IL-15 (*p* < 0.0001), IL-18 (*p* < 0.0001), IP-10 (*p* < 0.0001; Fig. 6H), M-CSF (*p* < 0.0001), MIP-3β (*p* < 0.0001; Fig. 6I), myostatin (*p* < 0.0001), OPG (*p* < 0.0001), sFas (*p* < 0.0001; Fig. 6J), TGFα (*p* < 0.0001), TRAIL (*p* < 0.0001; Fig. 6K), TSLP (*p* < 0.0001; Fig. 6L), IL-9 (*p* < 0.001), IL-4 (*p* < 0.01), IL-5 (*p* < 0.01), IL-8 (*p* < 0.01; Fig. 6F), IL-6 (*p* < 0.05), and MCP-1 (*p* < 0.05). Figure 7 shows changes over time in CSF levels of significantly different inflammatory markers, which remain consistently higher in patients with SCI + TBI.

**FIG. 8.**
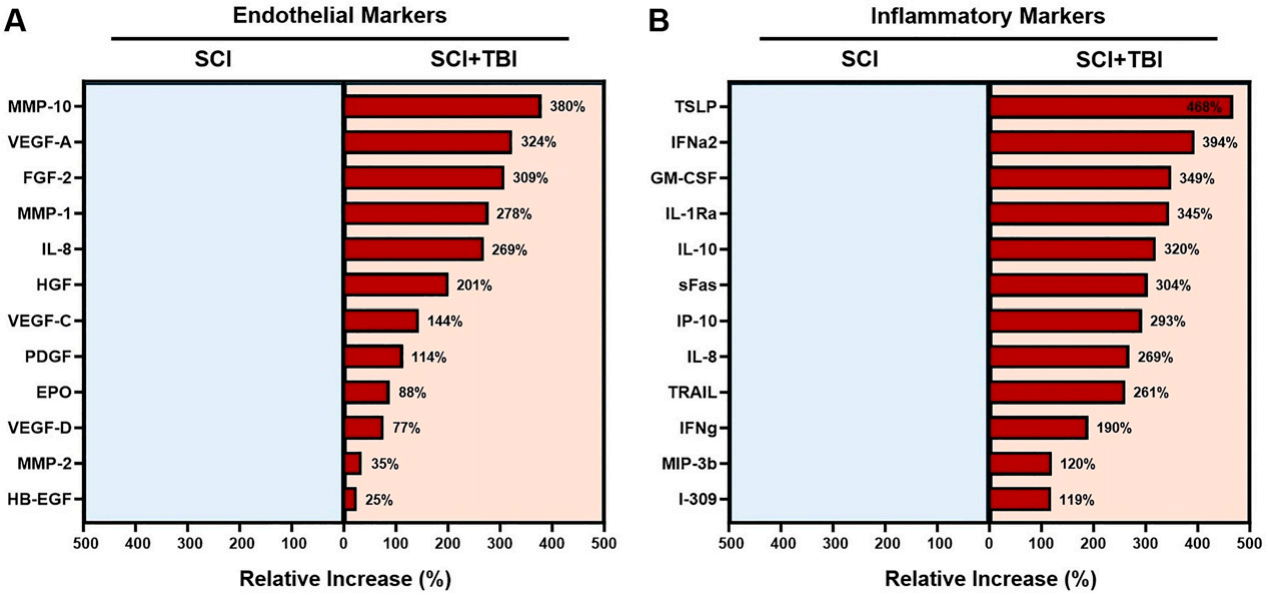
Relative increase (%) in adjusted/normalized mean values of endothelial **(A)** and inflammatory **(B)** markers in CSF of patients with SCI + TBI and those with isolated SCI. CSF, cerebrospinal fluid; EPO, erythropoietin; FGF, fibroblast growth factor; GM-CSF, granulocyte-macrophage colony-stimulating factor; HB-EGF, heparin-binding epidermal growth factor-like growth factor; HGF, hepatocyte growth factor; IFN, interferon; IL-1Ra, interleukin-1 receptor antagonist; IL-8, interleukin 8; IP, interferon gamma inducible protein; MIP, macrophage inflammatory protein; MMP, matrix metalloproteinase; PDGF-AA, platelet-derived growth factor AA; SCI, spinal cord injury; sFas, soluble Fas; TBI, traumatic brain injury; TRAIL, tumor necrosis factor-related apoptosis-inducing ligand; TSLP, thymic stromal lymphopoietin; VEGF, vascular endothelial growth factor.

Our analyses revealed that, overall, MMP-10 (380%), VEGF-A (324%), FGF-2 (309%), and MMP-1 (278%) exhibited the highest average increase in CSF levels in patients with SCI + TBI compared with those with SCI alone (Fig. 8A). Among inflammatory markers, TSLP, with an average increase of 468% in the SCI + TBI group, was the most differentially expressed factor, followed by IFNα2 (394%), GM-CSF (349%), IL-1Ra (345%), IL-10 (320%), sFas (304%), IP-10 (293%), and IL-8 (269%) (Fig. 8B). No endothelial or inflammatory marker showed an average relative increase in patients with SCI compared to patients with SCI + TBI.

**FIG. 9.**
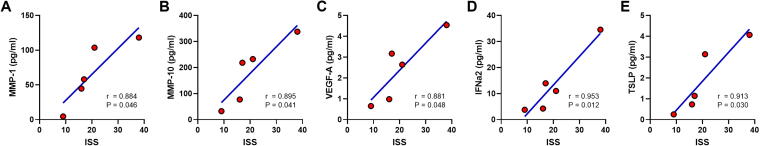
Correlations between ISS and MMP-1 **(A)**, MMP-10 **(B)**, VEGF-A **(C)**, IFNα2 **(D)**, and TSLP **(E)**. IFN, interferon; ISS, injury severity score; MMP, matrix metalloproteinase; TSLP, thymic stromal lymphopoietin; VEGF, vascular endothelial growth factor.

Interestingly, ISS correlated with average CSF levels of MMP-1 (r = 0.884 and *p* = 0.046; Fig. 9A), MMP-10 (r = 0.895 and *p* = 0.041; Fig. 9B), VEGF-A (r = 0.881 and *p* = 0.048; Fig. 9C), IFNα2 (r = 0.953 and *p* = 0.012; Fig. 9D), and TSLP (r = 0.913 and *p* = 0.030; Fig. 9E). No significant correlations were found between ISS and other CSF endothelial biomarkers or inflammatory mediators.

**FIG. 10.**
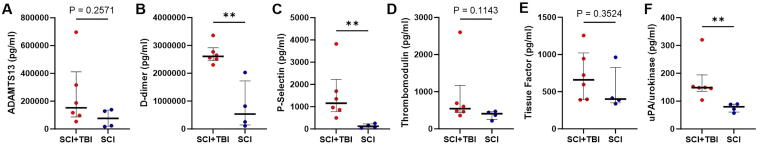
CSF levels of coagulation markers, including ADAMTS13 **(A)**, D-dimer **(B)**, P-selectin **(C)**, Thrombomodulin **(D)**, Tissue Factor **(E)**, and uPA/urokinase **(F)**. ADAMTS13, a disintegrin and metalloproteinase with thrombospondin motifs 13; CSF, cerebrospinal fluid; SCI, spinal cord injury; TBI, traumatic brain injury; uPA, urokinase-type plasminogen activator.

Analysis of coagulation markers showed that CSF levels of D-dimer (*p* < 0.01; Fig. 10B), P-selectin (*p* < 0.01; Fig. 10C), and uPA/urokinase (*p* < 0.01; Fig. 10F) were significantly higher in the SCI + TBI group compared with the SCI cohort. However, there were no significant differences in CSF ADAMTS13 (Fig. 10A), thrombomodulin (Fig. 10D), and tissue factor (Fig. 10E).

## Discussion

Trauma can cause both local and systemic endothelial dysfunction.^13^ Local endothelial dysfunction after neurotrauma, particularly TBI, is characterized by the breakdown of the BBB, leading to increased vascular leakage and triggering cytokine and chemokine release.^14^ This cascade may contribute to localized tissue edema and coagulation disturbances, and activate a local and/or systemic immune response, ultimately resulting in secondary CNS injury.^14^ Up to three-quarters of patients with SCI also experience some degree of TBI, and when TBI is present, it is associated with worse outcomes and increased mortality.^15–17^ However, it remains unclear whether TBI exacerbates endothelial dysfunction and damage in this group of patients. Therefore, we aimed to assess the impact of the type of CNS trauma on local endothelial dysfunction and to evaluate CSF levels of endothelial injury-associated biomarkers following different types of neurotrauma. Our findings help identify potential targets for therapeutic interventions aimed at preventing or mitigating the consequences of local endothelial disruption in patients with traumatic injury to the brain or spinal cord.

In response to endothelial damage and dysfunction caused by TBI or SCI, angiogenesis is essential for neuronal tissue repair and for reducing the impact of secondary injuries, such as hypoperfusion, hypoxia, and edema.^18,19^ Following neurotrauma, angiogenic growth factors, as markers of endothelial injury, are upregulated in the blood and CSF to re-establish adequate perfusion and restore the blood flow required for neurogenesis.^18,19^ Pro-angiogenic factors, including VEGFs and FGFs, particularly VEGF-A and FGF-2, promote endothelial cell proliferation and migration and minimize the BBB or BSCB disruption.^20–22^ Similarly, we observed that, overall, VEGF-A and FGF-2 showed the highest average increase among angiogenic markers in CSF in patients with SCI + TBI compared with those with isolated SCI. In addition, a significant correlation was found between CSF levels of VEGF-A and ISS, an indicator of overall injury severity. The dynamic changes over time in the expression of angiogenic factors after neurotrauma-induced endothelial injury dictate the pace and efficiency of the recovery process. In the early phase post-injury, elevated levels of these proteins, which start to upregulate within hours, are beneficial for healing and axonal regeneration.^23^ However, if levels remain excessively high for an extended period, they may lead to dysregulated angiogenesis, increased vascular leakage, and subsequent edema, which can worsen clinical outcomes.^23,24^ Thus, modulating key endothelial injury-related factors such as VEGF with proper timing and dosage presents a therapeutic target to optimize the angiogenic response, thereby enhancing healing and minimizing detrimental effects.^25,26^

Following neurotrauma, MMPs play important dual roles.^27^ On one hand, they possess proteolytic activity that can enhance angiogenesis by degrading the extracellular matrix, thereby facilitating endothelial cell migration and new blood vessel formation. Alternatively, MMPs contribute to the breakdown of the endothelial glycocalyx and the dysfunction of the BBB or BSCB.^27^ The glycocalyx, a gel-like layer coating the luminal side of endothelial cells, acts as a mechanical barrier, regulates vascular permeability, and modulates inflammation and coagulation through its interactions with blood components.^28^ Trauma-induced MMP-mediated glycocalyx shedding results in increased circulating components of the glycocalyx, such as syndecan-1, which correlates with poorer clinical outcomes.^29,30^ Several studies have documented the upregulation of MMPs both locally and systemically after traumatic injury of CNS.^31,32^ In response to TBI or SCI, MMP-1, MMP-3, MMP-9, and MMP-10 rapidly increase within hours post-injury, reflecting their role in acute glycocalyx degradation and barrier breakdown.^33,34^ This initial response is typically followed by a subsequent elevation of MMP-2, which is involved in tissue remodeling and repair and remains elevated for a longer period.^35,36^ Similarly, we found that CSF MMP-2 levels were higher in patients with SCI + TBI than those with SCI alone. In addition, patients with SCI + TBI had higher levels of MMP-1 and MMP-10, but not MMP-3 and MMP-9, compared with those with isolated SCI. Overall, in the presence of TBI, CSF MMP-10 and MMP-1 exhibited the greatest increases and also showed significant correlation with ISS, indicating their potential utility as biomarkers of injury burden. Beyond their primary functions, MMPs also facilitate immune cell migration and activate cytokines, modulating the immune response and inflammation, which may worsen secondary injuries following neurotrauma.^27^

The local immune response following both TBI and SCI exhibits temporal patterns that contribute to the inflammatory cascade, endothelial damage, barrier dysfunction, and secondary injuries.^37,38^ In both TBI and SCI, the early immune response to injury involves the activation of resident microglia and astrocytes, leading to the release of pro-inflammatory mediators.^39,40^ This early surge in cytokines and chemokines promotes the recruitment and activation of peripheral immune cells, predominantly neutrophils.^41,42^ Microglia and monocyte-derived macrophages, which initially exhibit a pro-inflammatory state, gradually shift toward an anti-inflammatory phenotype, aiding in tissue remodeling and repair.^37,40^ The cytokine and chemokine profiles reflect the temporal dynamics of cellular response to neurotrauma. In the present study, we evaluated CSF levels of inflammatory mediators, which more accurately reflect CNS inflammation than serum levels. Our analysis demonstrated that, among pro-inflammatory mediators, TSLP and IFNα2 were the most differentially expressed between SCI + TBI and SCI groups, with higher levels observed in patients with SCI+TBI. Notably, both also showed significant correlation with ISS, used to assess trauma severity in patients with multiple injuries. TSLP has been shown to peak early after TBI, while IFNα has been identified as a potential therapeutic target for both TBI and SCI.^43,44^ In addition to IFNα2, CSF levels of IFNγ and IP-10 were considerably higher in patients with SCI + TBI. IFNγ and IP-10 are functionally linked and closely related in the immune system.^44^ IFNγ drives a Th1-type immune response, promotes macrophage activation, and stimulates IP-10 production. IP-10, in turn, attracts inflammatory cells to the site of injury, further amplifying inflammation and thereby contributing to secondary endothelial damage.^45^ Our analysis also revealed that IL-8, a marker of both endothelial activation and inflammation, showed a marked increase in patients with SCI + TBI. Previous studies have reported that higher levels of CSF IL-8 are associated with more severe BBB disruption and increased mortality from TBI.^46,47^ Interestingly, we identified IL-10, an anti-inflammatory cytokine, to have one of the highest overall percentage increases in CSF levels in SCI + TBI cases compared with patients with SCI alone. This finding underscores the critical role of anti-inflammatory mediators in regulating inflammation after TBI and SCI. IL-10 downregulates pro-inflammatory cytokines and suppresses inflammatory cellular response.^48^ This negative feedback mechanism controls excessive inflammation, limits tissue damage, and promotes repair and regeneration.

Coagulation disturbances after neurotrauma, particularly TBI, may lead to secondary CNS injuries and worsened clinical outcomes.^9,49^ However, the interaction between the coagulation system and the vascular endothelium, as well as the mechanisms underlying hemostatic abnormalities, ranging from hypercoagulable to hypocoagulable states, remains poorly understood. TBI-induced coagulopathy typically presents as an initial hypercoagulation state, triggered by the release of brain-derived cellular microvesicles and activation of tissue factor, which can rapidly transition to a hypocoagulation state due to fibrinolysis and consumption coagulopathy, resembling disseminated intravascular coagulation.^9,49,50^ Similarly, we observed a significant increase in CSF levels of D-dimer in patients with SCI + TBI, indicating thrombosis and subsequent fibrinolysis, as well as elevated levels of P-selectin and urokinase, suggesting a pro-thrombotic condition and hyperfibrinolysis, respectively.

The main limitation of our study is the small patient cohort from a single center, which reflects the inherent challenges of obtaining longitudinal CSF samples in acute settings from patients with isolated SCI or combined SCI and TBI. This study was also limited by the lack of isolated TBI and healthy control groups. However, CSF from patients with isolated TBI is typically obtained via a ventriculostomy tube or an external ventricular drain rather than the lumbar drainage used in patients with SCI. This could introduce the potential for site-dependent variability due to loco-regional differences in biomarker levels, limiting direct comparisons. In addition, collecting CSF from healthy individuals at multiple time points would be ethically and logistically impractical. Another limitation was the lack of blood samples; however, blood levels may not accurately reflect local injury to the CNS.

## Conclusion

The presence of TBI in patients with SCI was associated with the highest relative increases in CSF levels of the endothelial biomarkers MMP-10, VEGF-A, and FGF-2, and inflammatory mediators TSLP, IFNα2, and GM-CSF. Furthermore, CSF levels of MMP-1, MMP-10, VEGF-A, IFNα2, and TSLP showed significant correlation with ISS, highlighting their potential clinical relevance. Our findings identify several promising therapeutic targets, such as angiogenic factors and MMPs, for reducing endothelial injury and preventing neurovascular damage in patients with TBI and SCI. In addition, CSF biomarkers of endotheliopathy may serve as prognostic indicators to identify patients at high risk of poor clinical outcomes and to guide early, personalized interventions. Future studies involving larger, multi-group cohorts are needed to further validate and expand upon these findings and to evaluate therapeutic strategies aimed at mitigating secondary injury and improving outcomes in neurotrauma patients.

## Abbreviations Used

ADAMTS13: a disintegrin and metalloproteinase with thrombospondin motifs 13
AIS: abbreviated injury scale
BBB: blood-brain barrier
BSCB: blood-spinal cord barrier
CNS: central nervous system
CSF: cerebrospinal fluid
EPO: erythropoietin
FGF: fibroblast growth factor
FLT3: fms-like tyrosine kinase 3
GM-CSF: granulocyte-macrophage colony-stimulating factor
HB-EGF: heparin-binding epidermal growth factor-like growth factor
HGF: hepatocyte growth factor
IFN: interferon
IL: interleukin
IL-1Ra: interleukin 1 receptor antagonist
IP-10: interferon gamma inducible protein 10
ISS: injury severity score
MCP-1: monocyte chemoattractant protein 1
M-CSF: macrophage colony-stimulating factor
MIP-3β: macrophage inflammatory protein 3β
MMP: matrix metalloproteinase
OPG: osteoprotegerin
PCA: principal component analysis
PDGF: platelet-derived growth factor
SCI: spinal cord injury
sFas: soluble Fas
TBI: traumatic brain injury
TGFα: transforming growth factor α
TRAIL: tumor necrosis factor-related apoptosis-inducing ligand
TSLP: thymic stromal lymphopoietin
uPA: urokinase-type plasminogen activator
VEGF: vascular endothelial growth factor
